# FOXC1 Regulation of miR-31-5p Confers Oxaliplatin Resistance by Targeting LATS2 in Colorectal Cancer

**DOI:** 10.3390/cancers11101576

**Published:** 2019-10-16

**Authors:** Hsi-Hsien Hsu, Wei-Wen Kuo, Hui-Nung Shih, Sue-Fei Cheng, Ching-Kuo Yang, Ming-Cheng Chen, Chuan-Chou Tu, Vijaya Padma Viswanadha, Po-Hsiang Liao, Chih-Yang Huang

**Affiliations:** 1 Division of Colorectal Surgery, Department of Surgery, MacKay Memorial Hospital, Taipei 251, Taiwan; hsu5936@mmh.org.tw (H.-H.H.); yangchingkao@yahoo.com.tw (C.-K.Y.); 2 MacKay Medicine, Nursing and Management College, Taipei 104, Taiwan; 149374@tahsda.org.tw; 3 Department of Biological Science and Technology, China Medical University, Taichung 404, Taiwan; wwkuo@mail.cmu.edu.tw; 4 Medical Research Center for Exosome and Mitochondria Related Diseases, China Medical University and Hospital, Taichung 404, Taiwan; a99nita32@yahoo.com.tw (H.-N.S.); robert750927@hotmail.com (P.-H.L.); 5 Department of Pharmacy, Taiwan Adventist Hospital, Taipei 105, Taiwan; 6 Faculty of Medicine, National Yang-Ming University, Taipei 112, Taiwan; claudiochen7@gmail.com; 7 Division of Colorectal Surgery, Taichung Veterans General Hospital, Taichung 407, Taiwan; 8 Division of Chest Medicine, Department of Internal Medicine, Armed Force Taichung General Hospital, Taichung 411, Taiwan; tu4697@gmail.com; 9 Department of Biotechnology, Bharathiar University, Coimbatore-641 046, India; vvijayapadma@rediffmail.com; 10 Graduate Institute of Biomedicine, China Medical University and Hospital, Taichung 404, Taiwan; 11 Division of General Surgery, Department of Surgery, Shuang Ho Hospital, Taipei Medical University, New Taipei City 235, Taiwan; 12 Cardiovascular and Mitochondrial Related Disease Research Center, Hualien Tzu Chi Hospital, Buddhist Tzu Chi Medical Foundation, Hualien 970, Taiwan; 13 Center of General Education, Buddhist Tzu Chi Medical Foundation, Tzu Chi University of Science and Technology, Hualien 970, Taiwan; 14 Graduate Institute of Biomedical Sciences, China Medical University, Taichung 404, Taiwan; 15 Department of Medical Research, China Medical University Hospital, China Medical University, Taichung 404, Taiwan; 16 Department of Biotechnology, Asia University, Taichung 413, Taiwan

**Keywords:** colorectal cancer (CRC), drug-resistance mechanism, oxaliplatin, microRNAs, large tumor suppressor kinase 2 (LATS2), Forkhead box C1 (FOXC1)

## Abstract

Colorectal cancer (CRC) is the second leading cause of cancer-related illness worldwide and one of the most common malignancies. Therefore, colorectal cancer research and cases have gained increasing attention. Oxaliplatin (OXA) is currently used in first-line chemotherapy to treat stage III and stage IV metastatic CRC. However, patients undergoing chemotherapy often develop resistance to chemo drugs being used. Evidence has confirmed that microRNAs regulate downstream genes in cancer biology and thereby have roles related to tumor growth, proliferation, invasion, angiogenesis, and multi-drug resistance. The aim of our study is to establish whether miR-31-5p is an oncogene in human colorectal cancers that are resistant to OXA and further confirm its malignant phenotype-associated target molecule. From the results of miRNA microarray assay, we establish that miR-31-5p expression was upregulated in oxaliplatin-resistant (OR)-LoVo cells compared with parental LoVo cells. Moreover, through in vitro and in vivo experiments, we demonstrate that miR-31-5p and large tumor suppressor kinase 2 (LATS2) were inversely related and that miR-31-5p and Forkhead box C1 (FOXC1) were positively correlated in the same LoVo or OR-LoVo cells. Importantly, we reveal a novel drug-resistance mechanism in which the transcription factor FOXC1 binds to the miR-31 promoter to increase the expression of miR31-5p and regulate LATS2 expression, resulting in cancer cell resistance to OXA. These results suggest that miR-31-5p may be a novel biomarker involved in drug resistance progression in CRC patients. Moreover, the FOXC1/miR31-5p/LATS2 drug-resistance mechanism provides new treatment strategies for CRC in clinical trials.

## 1. Introduction

Colorectal cancer (CRC) is the second leading cause of cancer-related illness worldwide and one of the most common malignancies [1]. It is also the number one cause of cancer mortality in Taiwan [2,3,4]. CRC is the third leading cause of death among cancers, and the number of deaths due to CRC is higher in males than in females in Taiwan [5,6]. Colorectal carcinoma patients’ symptoms include changes in bowel habits, weakness or fatigue, unexplained weight loss, and an abnormal lifestyle. CRC patients are usually treated with surgery, radiation, chemotherapy, targeted drugs, or a combination of these therapies [7]. CRC patients with early-stage disease have 5-year survival rates greater than 90%. However, approximately 40% or less develop stage IV metastasis [8,9].

Oxaliplatin (OXA) is a third-generation drug employed for first-line chemotherapy of CRC and has been used to treat stage III and stage IV colorectal cancer. An increasing number of reports concerning OXA resistance in CRC treatment have suggested that resistance is an urgent issue in clinical applications [10,11,12,13].

MicroRNAs (miRNAs) are short RNAs that contain approximately 20–22 nucleotides [14]. MiRNAs bind to the 3’untranslated region (3’UTR) of their target mRNAs. Perfect complementarity with their target mRNAs causes mRNA deadenylation and degradation, and imperfect complementarity causes translational inhibition of mRNA and consequently reduces protein expression. A growing body of literature has demonstrated the importance of miRNAs in tumor pathogenesis, progression, and the therapeutic response, and these molecules have been defined as potential cancer biomarkers [7,15,16,17,18,19,20,21]. 

Several investigators have reported that miR-31 functions not only as an oncogene but also as a tumor suppressor in specific types of cancers. Upregulated miR-31 expression has been identified in lung cancer, colorectal cancer, and endometrial cancer, whereas it plays a tumor-suppressive role in breast cancer, gastric cancer, ovarian cancer, malignant mesothelioma, and pancreatic cancer [22,23,24,25,26,27]. The functional and regulatory activities of miRNA-31 regarding the clinical prognostic significance of CRC are not fully understood. 

The literature has shown that miRNA-650 is able to serve as an oncogene and promote cell proliferation by directly targeting large tumor suppressor kinase 2 (LATS2) in non-small cell lung cancer formation and progression [28]. MiR-135b was shown to regulate CRC cell proliferation, apoptosis, and chemoresistance by negatively regulating LATS2 expression. LATS2 plays a central role in the mediation of Hippo growth-inhibitory signaling, and it can regulate mitotic progression, YAP activation, retinoblastoma protein (pRB) activity, and p53 activity, leading to cell cycle arrest and inhibition of tumor growth [29,30,31,32,33,34]. 

Our previous research has shown that the proliferation rate of oxaliplatin-resistant (OR)-LoVo cells was higher than that of parental LoVo cells. OR-LoVo cells overcome OXA-induced G2/M phase cell cycle arrest [10]. In this study, we aim to clarify the role of miRNA in the drug resistance mechanism in an OXA-resistant CRC cell model. Our data show that the upregulated expression of FOXC1 and miR-31-5p inhibited LATS2 expression and led to CRC cell resistance to OXA. Moreover, the knockdown of miR-31-5p resulted in cancer cell apoptosis, decreased cell proliferation, and enhanced chemosensitivity. These results were further confirmed in a xenograft animal model. In this study, we demonstrate a novel drug-resistance mechanism in which miR-31-5p and its transcription factor regulate cancer cell growth and apoptosis by targeting LATS2. Our findings suggest that miR-31-5p may be considered as a biomarker and new therapeutic target in the progression of drug resistance in CRC cancer patients.

## 2. Results

### 2.1. Evaluation of the Cell Properties of LoVo and OR-LoVo Cells

**Figure 1:**
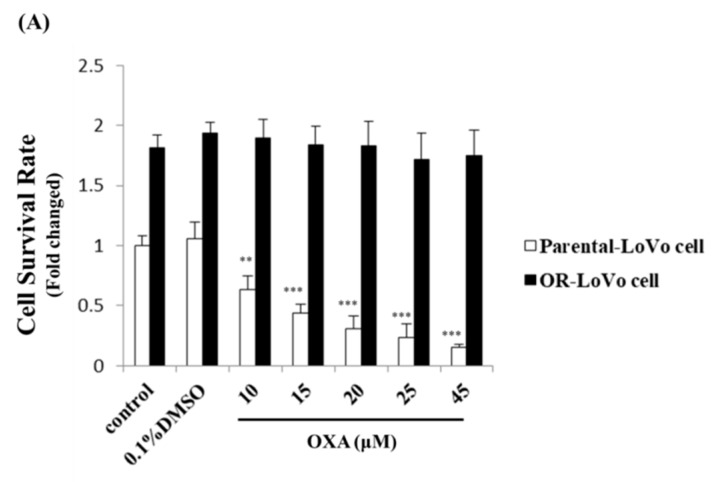

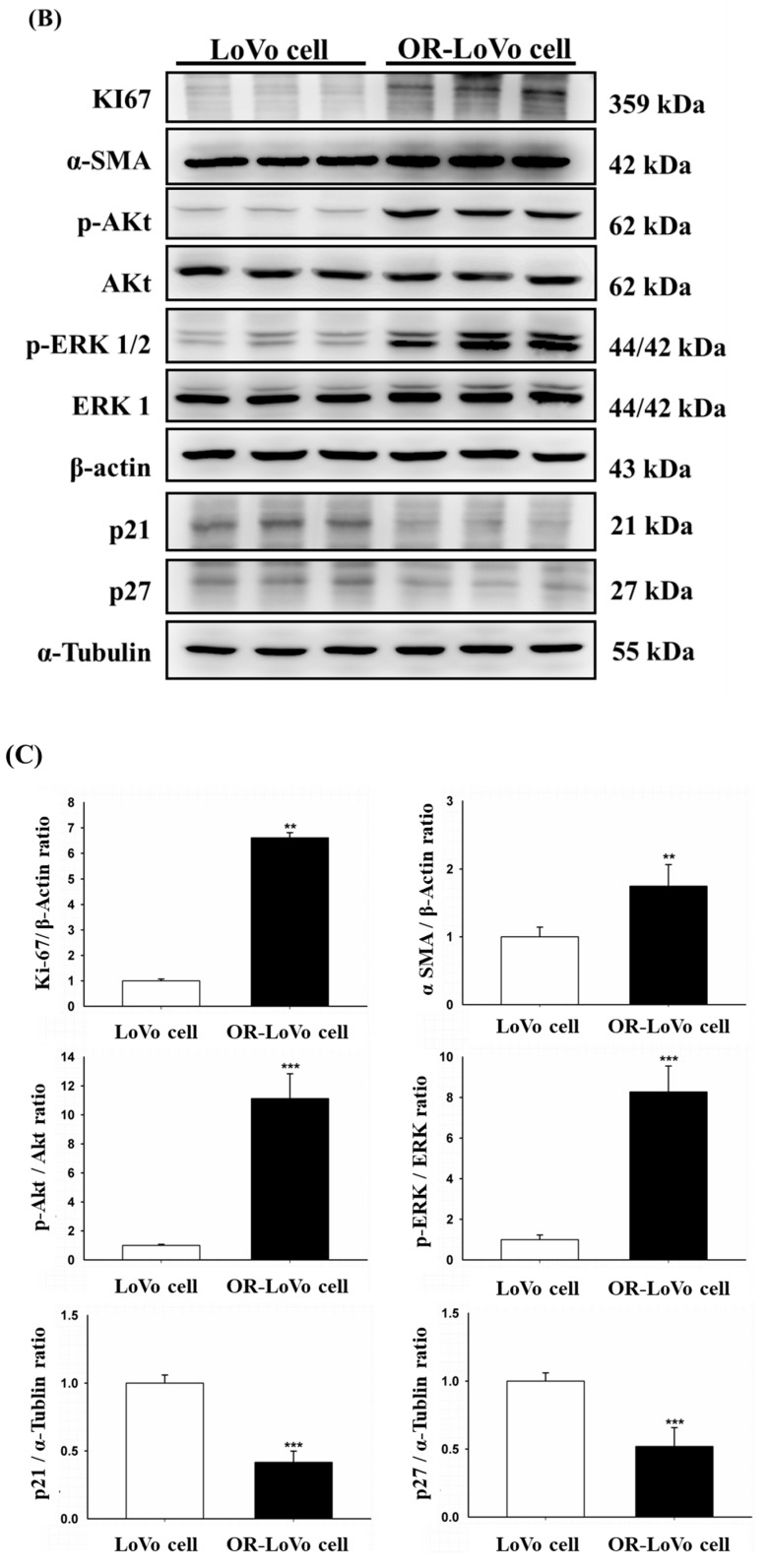
Cell properties differ between LoVo cells and oxaliplatin resistance (OR)-LoVo cells. (**A**) The MTT assay result indicates the survival rate of OR-LoVo cells after treatment with or without oxaliplatin (OXA) (45 μM at 24 h) compared with the LoVo cell control group. ** *p* < 0.01 vs. the LoVo cell control group; *** *p* < 0.001 vs. the LoVo cell control group. (**B**) Expression of cell proliferation- and cell cycle checkpoint proteins in LoVo cells and OR-LoVo cells by Western blotting. (**C**) Quantification of the protein expression of Ki-67, α-SMA, p-Akt, p-ERK, p21, and p27 (*n* = 3). ** *p* < 0.01 vs. LoVo cells; *** *p* < 0.001 vs. LoVo cells.

In this study, we used oxaliplatin (OXA) that was changed from IV-injected liquid medicine (Sanofi, Paris, France; obtained from Dr. Ming-Cheng Chen [10]) to the purified drug (Sigma, St. Louis, MO, USA) and followed a previous protocol [10] to establish the OR-LoVo cell line. Figure 1A shows that IC_50_ in LoVo cells was 15 μM, whereas IC_50_ in OR-LoVo cells was 3-fold (45 μM) that of parental cells. Next, we also confirmed that characteristics differ between LoVo and OR-LoVo cells. The MTT assay result indicates that the cell viability of OR-LoVo cells was higher than that of LoVo cells for each control group, with OR-LoVo cells having markedly increased cell proliferation at 24 h (Figure 1A). Moreover, we harvested the total protein from three different passages of the two cell lines to evaluate their expression of functional proteins related to cell proliferation, epithelial–mesenchymal transition (EMT), and cell cycle arrest by Western blotting assay. The results show that the levels of the cell proliferation-related proteins KI67, p-Akt, p-ERK, and the EMT marker α-SMA all significantly increased in OR-LoVo cells compared with their levels in LoVo cells. Further, the expression of the cell cycle checkpoint proteins p21 and p27 decreased in OR-LoVo cells compared with that in LoVo cells (Figure 1B,C). These data confirm the resistance of OR-LoVo cells, which can resist up to 45 μM OXA treatment and have higher proliferation compared with parental cells.

### 2.2. MicroRNA Expression Differed between LoVo and OR-LoVo Cancer Cells

**Figure 2:**
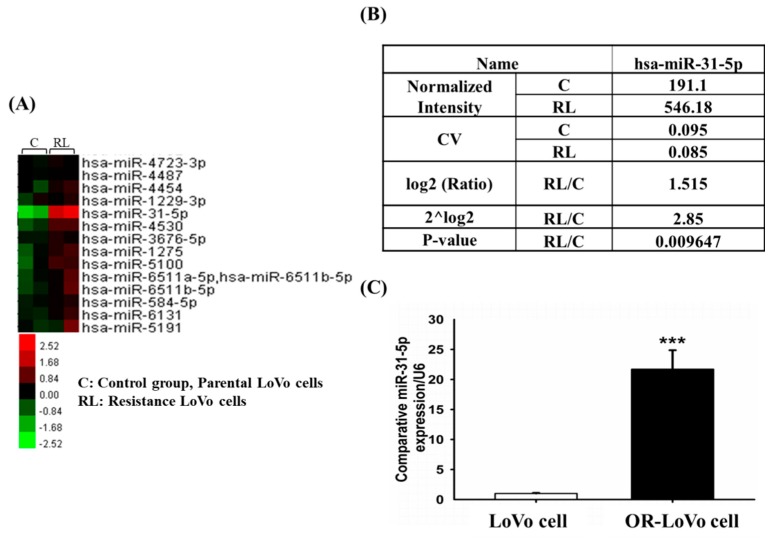
MicroRNA expression in LoVo and OR-LoVo cells. (**A**) MiRNA microarray data analysis, with the red bar indicating upregulated expression and the green bar indicating downregulated expression. (**B**) Detailed miRNA microarray data analysis lists the hsa-miR-31-5p C, RL, or RL/C (C is LoVo cells; RL is OR-LoVo cells) value. C is LoVo cells; RL is OR-LoVo cells. (**C**) Results of the qRT-PCR analysis of the expression levels of miR-31-5p are shown by the bar. *** *p* < 0.001 vs. LoVo cells.

Recent studies have shown that microRNA plays an important role in the regulation of tumor progression [35,36,37]. To follow up on these findings, we hypothesized that the acquired OXA resistance of OR-LoVo cells was not only related to changes in protein expression (Figure 1) but also highly correlated with microRNAs (miRNAs). We determined the expression of miRNAs by microarray assay. The result shows that miR-31-5p was one of the miRNAs whose expression differed between OR-LoVo cells and LoVo cells (Figure 2A). According to Figure 2A, the expression of miR-31-5p was upregulated in OR-LoVo cells compared with that in LoVo cells. Comparison of the raw data on hsa-miR-31-5p expression in the two cell lines show that RL/C (C is LoVo cells; RL is OR-LoVo cells) had a log2 value of 1.515 ≥ 0.8, 2^^log2^ value of 2.85, and *p* value of 0.009647 < 0.05 (Figure 2B). From the microarray data, we confirmed the miR-31-5p expression in the two cell lines by qPCR. The result indicates that miRNA-31-5p expression was significantly increased in OR-LoVo cells compared with that in LoVo cells (Figure 2C). These results show that miRNA expression differed between the two cell lines and that miR-31-5p may play an important role in LoVo cell resistance to OXA.

### 2.3. MiR-31-5p Regulates Cell Survival and Cell Death in LoVo and OR-LoVo Cells in Vitro

**Figure 3:**
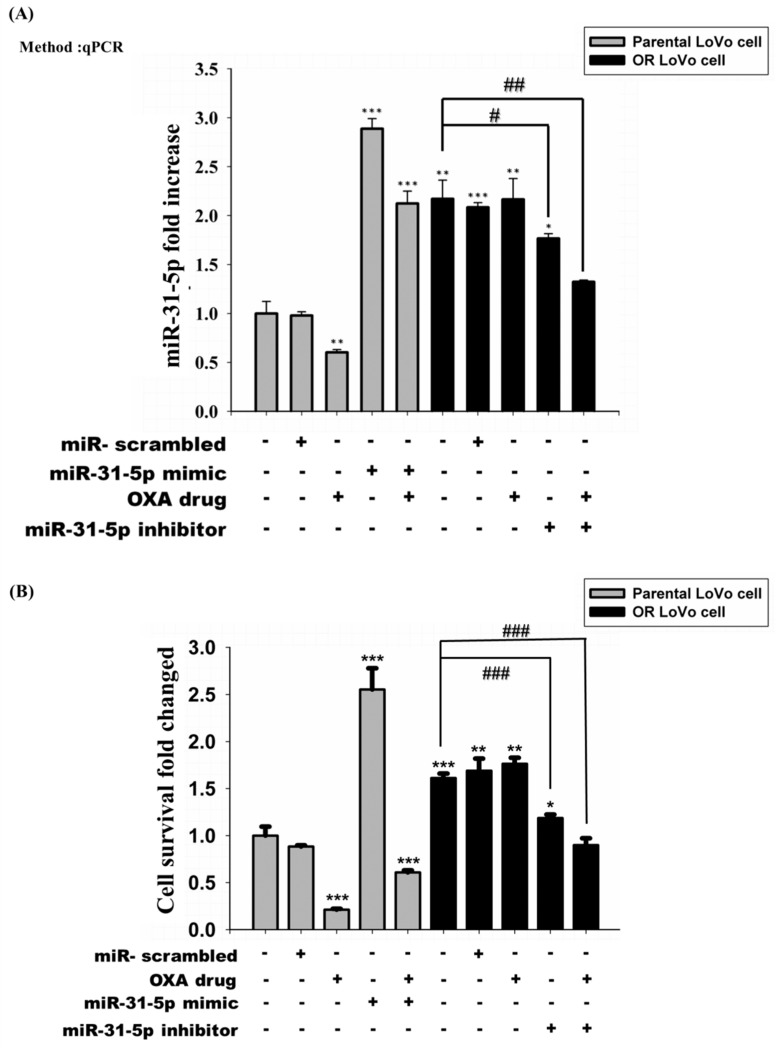

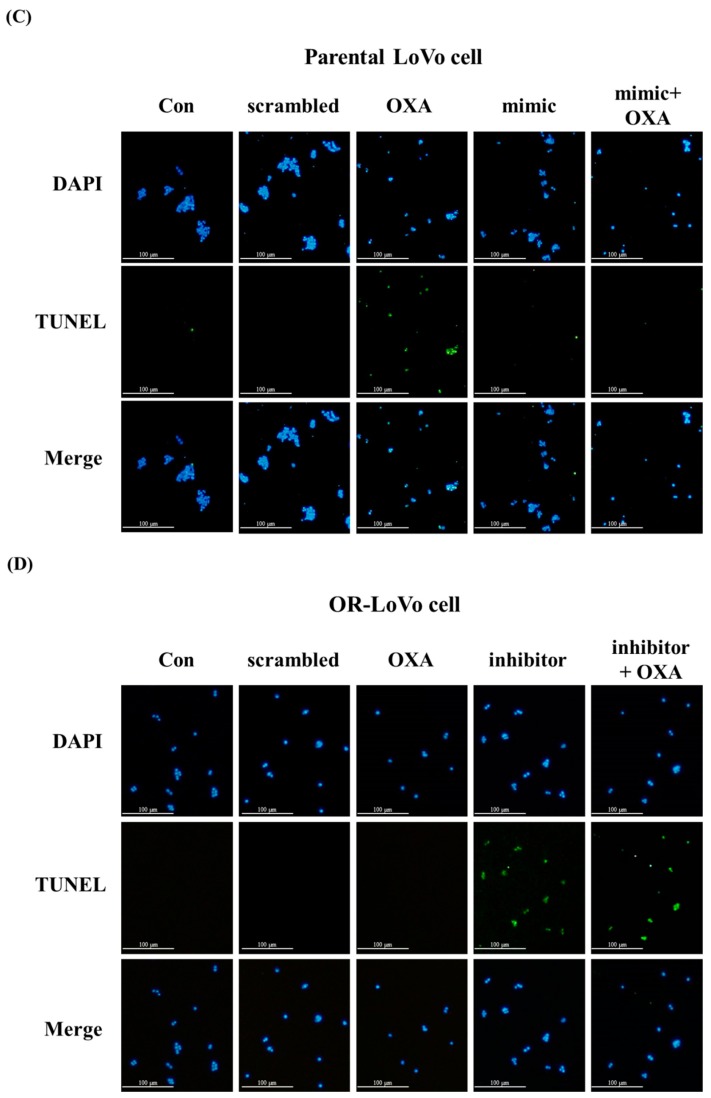
The effect of the overexpression or knockdown of miR-31-5p and treatment with or without OXA on cell survival and cell death of LoVo or OR-LoVo cells in vitro. (**A**) MiR-31-5p expression in LoVo or OR-LoVo cells transfected with a miR-31-5p mimic (40 nM), inhibitor (40 nM), or scrambled miRNA (40 nM) (mimic negative control (NC) or inhibitor NC) or treatment with OXA (45 μM). MiR-31-5p fold increase which was calculated by the ratio miR-31-5p/U6, being U6 snRNA constitutively expressed. (**B**) Cell survival fold changed of LoVo cells or OR-LoVo cells determined by the MTT assay. The TUNEL assay was used to detect cell death in LoVo cells (**C**) and OR-LoVo cells (**D**). Fluorescein staining was used to indicate apoptotic cells, and DAPI staining was used to determine the number of nuclei and assess the gross cellular morphology. LoVo_scrambled represents the miRNA mimic NC, LoVo_mimic represents the miR-31-5p mimic (overexpression of miR-31-5p), OR_scrambled represents the miRNA inhibitor NC, OR_inhibitor represents the miR-31-5p inhibitor (knockdown of miR-31-5p), and OXA represents oxaliplatin. * *p* < 0.05 vs. LoVo cells control group; ** *p* < 0.01 vs. LoVo cells control group; *** *p* < 0.001 vs. LoVo cells control group. # *p* < 0.05 vs. OR-LoVo cells control group; ## *p* < 0.01 vs. OR-LoVo cells control group; ### *p* < 0.001 vs. OR-LoVo cells control group.

Previous data show that the expression of miR-31-5p was higher in OR-LoVo cells than parental cells. We used a miR-31-5p mimic and inhibitor to examine the role of miR-31-5p in the two cell lines. Figure 3A shows that transfection with the miR-31-5p mimic and inhibitor to regulate the expression of miR-31-5p in the two cell lines was successful. The OXA treatment suppressed the expression of miR-31-5p but did not influence the transfection ability of the miR-31-5p mimic in LoVo cells. The miR-31-5p inhibitor was able to successfully suppress miR-31-5p expression in OR-LoVo cells and OXA-treated OR-LoVo cells. However, the expression of miR-31-5p in OR-LoVo cells did not decrease when treated with OXA only. Next, we used MTT and TUNEL assays to investigate the effects of miR-31-5p or OXA on the cell survival rate in the two cell lines. The MTT result shows that OR-LoVo cells had a higher proliferation rate than LoVo cells and were resistant to OXA treatment (Figure 3B). Moreover, the TUNEL assay results confirm that OXA induced apoptosis in LoVo cells but not in OR-LoVo cells (Figure 3C,D). Interestingly, OXA treatment suppressed miR-31-5p expression and also induced cell apoptosis in LoVo cells but not in OR-LoVo cells (Figure 3A). This result suggests that the expression of miR-31-5p may be highly related to LoVo cells’ resistance to OXA. Building on our previous results, we upregulated the overexpression of miR-31-5p by a mimic-induced increase in cell proliferation and cell viability in LoVo cells after treatment with OXA. In contrast, cell proliferation was suppressed after the knockdown of miR-31-5p by transfecting OR-LoVo cells with the miR-31-5p inhibitor. Importantly, the knocked down expression of miR-31-5p enhanced the chemosensitivity of OR-LoVo. Our data suggest that miR31-5p plays an important role in LoVo cell resistance to OXA by promoting cell growth and suppressing cell apoptosis. 

### 2.4. LATS2 is a Direct Target of miR-31-5p in Colorectal Cancer Cells

**Figure 4:**
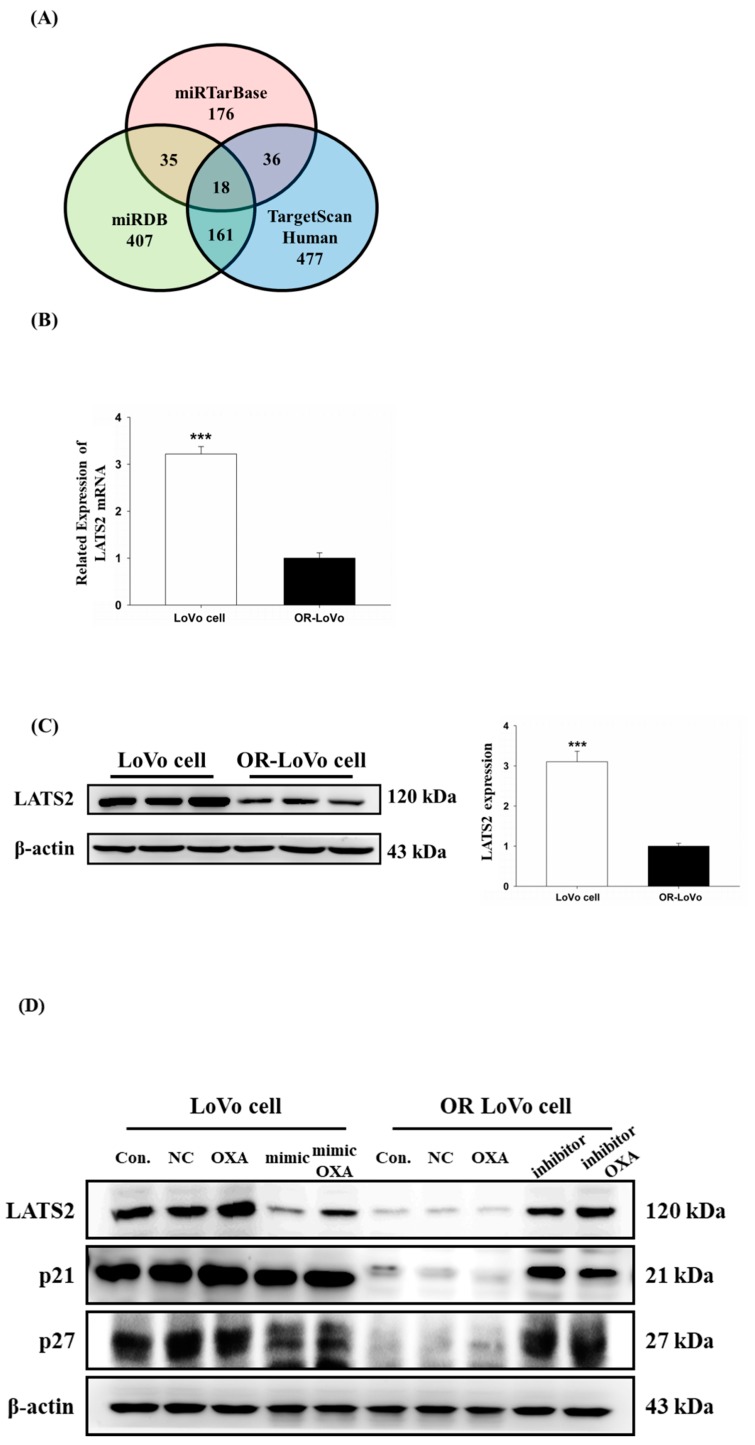

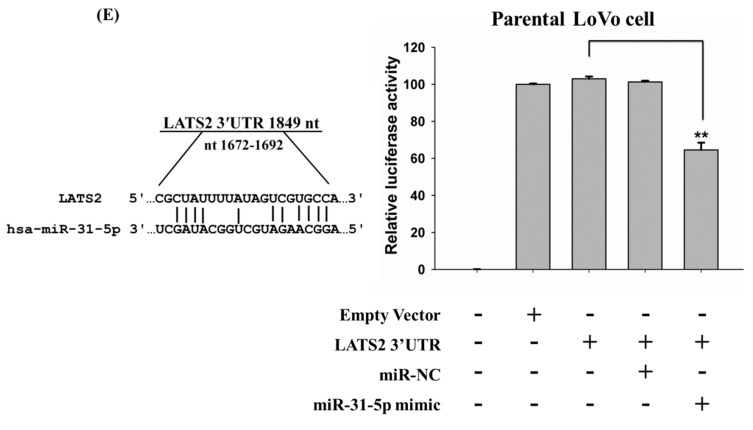
MiR-31-5p regulates LATS2 mRNA and protein expression by targeting the 3’UTR in LoVo cells and OR-LoVo cells. (**A**) We combined the miRTarBase, miRDB, and TargetScanHuman databases to predict the putative targets of miR-31-5p, and there were 18 predicted targets that overlapped among the three online databases. (**B**) Real-time RT-PCR analysis was used to confirm the mRNA expression levels of LATS2 in the two cell lines. *** *p* < 0.001 vs. OR-LoVo cells. (**C**) Protein expression of LATS2 in LoVo cells and OR-LoVo cells by Western blotting. Quantification of LATS2 protein expression (*n* = 3, the 3 lanes for each cell line loaded in the Western blot which were harvested from the total protein of the three different passages). *** *p* < 0.001 vs. OR-LoVo cells. (**D**) Protein expression of LATS2, p21, and p27 in LoVo or OR-LoVo cells transfected with the miR-31-5p mimic (40 nM), inhibitor (40 nM), or scrambled miRNA (40 nM) (mimic NC or inhibitor NC), or treatment with or without OXA (45 μM) by Western blotting. The experiments were performed in triplicate. (**E**) Luciferase activity assays of the activity of luciferase vectors containing the LATS2 3′-UTR were performed following transfection with miR-31-5p or negative control (NC) for 24 h using the Dual-Luciferase Reporter Assay System (Promega, Madison, WI, USA). ** *p* *<* 0.01 vs. LoVo cells LATS2 3’UTR group.

Our previous results show that miR-31-5p plays an important role in the resistance mechanism of colorectal cancer cells. Next, we aimed to find the direct target of miR-31-5p. We used three online databases—miRTarBase, miRDB, and TargetScanHuman—to predict the miR-31-5p targets (Figure 4A). Among these target genes, LATS2 was a predicted target that overlapped in the three databases and is highly related to tumorigenesis. Following this finding, we analyzed the mRNA and protein expression of LATS2 by quantitative RT-PCR, and Western blot in the two cell lines. We found that LATS2 mRNA expression decreased in OR-LoVo cells compared with parental cells by quantitative RT-PCR (Figure 4B). Moreover, we harvested the total protein from three different passages of the two cell lines for Western blotting assay. The result shows that the protein expression level of LATS2 was downregulated in OR-LoVo cells compared with that in parental cells (Figure 4C). Accordingly, we investigated whether miR-31-5p regulates LATS2 expression and the downstream molecular pathway by transfecting cells with a miR-31-5p mimic and inhibitor. Figure 4D indicates that the expression of LATS2 and the downstream genes p21 and p27 was significantly reduced in OR-LoVo cells compared with that in LoVo cells. After overexpression of miR31-5p, the expression of LATS2 and downstream proteins decreased in parental cells, whereas the knockdown of miR31-5p expression by the inhibitor significantly increased the expression of LATS2 and downstream proteins in resistant cells. Our data suggest that LATS2 expression is highly correlated with miR-31-5p, so we further confirmed that LATS2 is a direct target of miR-31-5p by luciferase assay. As shown in Figure 4E, the miR-31-5p binding sequence is located on the LATS2 3’UTR, which was constructed into a luciferase assay reporter vector (LATS2 3’UTR). After co-transfecting parental LoVo cells with the LATS2 3’UTR reporter vector and miR-31-5p mimic, the luciferase activity significantly decreased compared with the LATS2 3’UTR reporter vector alone.

Importantly, OXA activated the LATS2 pathway in parental cells, but this function was lost after co-treatment with the miR-31-5p mimic. In contrast, OXA could not activate the LATS2 pathway, but after co-treatment with the miR-31-5p inhibitor, the expression of the LATS2 pathway increased. These results suggest that miR31-5p may play an important role in LoVo cell resistance to OXA by targeting LATS2. These results indicate that miR-31-5p regulates colorectal cancer cell sensitivity to OXA by targeting LATS2.

### 2.5. MiR-31-5p Regulates Tumorigenesis and Chemosensitivity by Targeting LATS2 in A Xenograft Tumor Model

**Figure 5:**
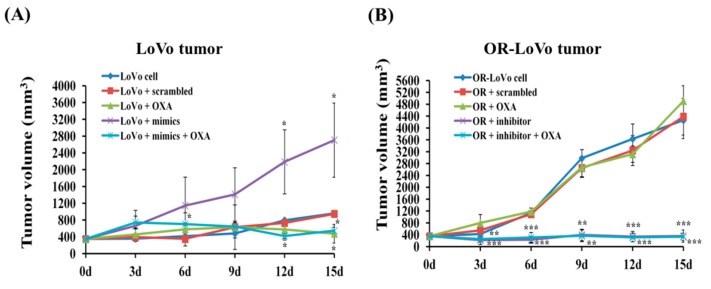

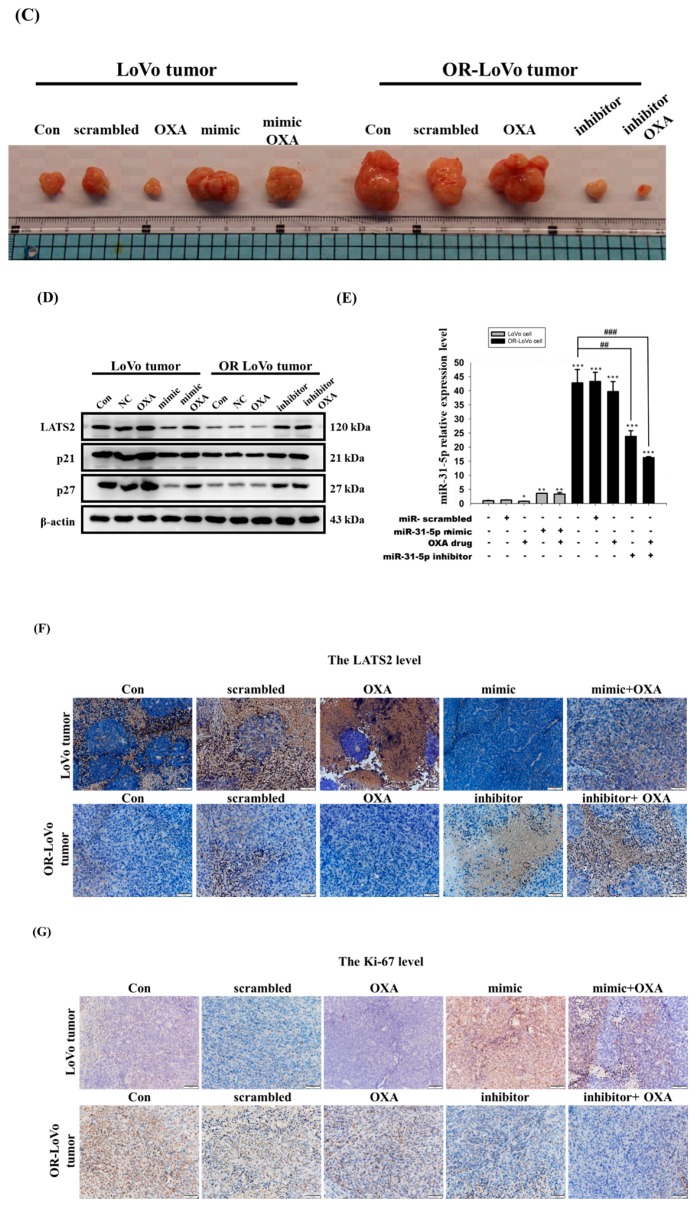

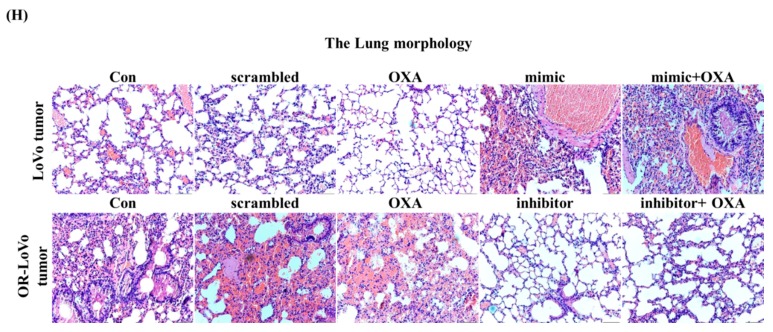
MiR-31-5p or OXA regulates tumor growth and tumor death in LoVo or OR-LoVo tumors in vivo. (**A**) The tumoral growth of LoVo cell xenografted on nude mice in five different groups. Treatment was administered every 3 days from day 0 to 15. (**B**) The tumoral growth of LoVo cell xenografted on nude mice in five different groups. Treatment was administered every 3 days from day 0 to 15. Tumors of LoVo or OR-LoVo cell were subcutaneously implanted. (**C**) Representative pictures of tumoral masses isolated at the moment of sacrifice of mice. The nude mice were sacrificed at day 15. (**D**) Expression of LATS2, p21, and p27 in LoVo and OR-LoVo tumor tissue by Western blotting. (**E**) MiR-31-5p expression in LoVo and OR-LoVo tumor tissue was measured by qRT-PCR. U6 was used as a loading control. Scale bar: 100 μm (**F**) IHC staining of LATS2 protein in LoVo or OR-LoVo tumor tissue samples. Scale bar: 100 μm. (**G**) IHC staining of Ki-67 protein in LoVo or OR-LoVo tumor tissue samples. Scale bar: 100 μm. (**H**) Lung metastasis in mice bearing LoVo and OR-LoVo cells. Upper panel: representative HE-stained sections of lungs from mice with colorectal cancer cell (CRC) metastasis. Scale bar: 100 μm. * *p* < 0.05 vs. vs. LoVo cells control group; ** *p* < 0.01 vs. LoVo cells control group; *** *p* < 0.001 vs. LoVo cells control group. ## *p* < 0.01 vs. OR-LoVo cells control group; ### *p* < 0.001 vs. OR-LoVo cells control group.

Our previous results indicate that miR-31-5p plays an important role in the drug resistance mechanism and cell activity in a cancer cell model. Our results also show that the mice in the in vivo experiment underwent significant weight loss in the LoVo + mimic group. Interestingly, the mice gained weight in the OR + inhibitor and OR + inhibitor + OXA groups (Supplementary Figure S1A,B). These results indicate that the expression of miR-31-5p is related to tumor-induced body weight loss and sensitivity to OXA. When tumor growth reached 300–400 mm^3^, we treated each mouse with the mimic, inhibitor, or OXA. We found that treatment with OXA inhibited tumor growth, but the miR-31-5p mimic promoted tumor growth in a time-dependent manner compared with the control group (Figure 5A). Moreover, after tumors were injected with the miR31-5p inhibitor, tumor volume was reduced, and tumor sensitivity to OXA was enhanced (Figure 5B). The results in Figure 5E confirm that the miR-31-5p mimic promoted LoVo tumor growth and reduced its sensitivity to OXA. Moreover, the miR-31-5p inhibitor reduced the tumor size and promoted sensitivity in cells resistant to OXA. These data suggest that miR-31-5p induces tumor proliferation and modulates sensitivity to OXA drug chemotherapy. 

After isolated tumor tissue from nude mice, we can see the tumor growth was faster in OR-LoVo control group compared with LoVo control group and the tumor size changed related with miR-31-5p expression (Figure 5C). Next, we targeted LATS2 in tumor tissue to investigate whether miR-31-5p is involved in the mediation of OXA resistance and regulation of cancer growth. Western blot data show that the expression of LATS2 and the downstream genes p21 and p27 was higher in LoVo cells compared with resistant cells. After treatment with OXA, LATS2 pathway expression was increased in parental cells but not in resistant cells. Moreover, the miR-31-5p mimic inhibited the LATS2 pathway expression and promoted tumor growth in LoVo cells. However, treatment with the miR-31-5p inhibitor induced LATS2, p21, and p27 protein expression in OR-LoVo cells (Figure 5D). Importantly, our data show that miR-31-5p not only regulated its target protein but also affected the OXA-induced LATS2 pathway activation in the two cell lines. These results indicate that miR-31-5p is highly related to LoVo cells’ resistance to OXA. We used qRT-PCR to analyze miR-31-5p expression after treatment with OXA, the mimic, or the inhibitor in two different tumors (Figure 5E). 

The immunohistochemistry (IHC) data support the previous results and indicate that the expression of LATS2 contrasted miR-31-5p expression (Figure 5F). Moreover, we also sought to confirm whether miR31-5p regulates cell proliferation in vitro. We further analyzed tumor proliferation by staining tumor sections to detect Ki-67. The Ki-67 protein level in the OR-LoVo control group was higher than that in the LoVo control group. Transfection with the mimic to overexpress miR-31-5p led to Ki-67 upregulation in parental cells, and the knockdown of miR-31-5p in OR-LoVo decreased the Ki-67 protein level compared with that in the control group (Figure 5G). Importantly, the overexpression of miR-31-5p decreased OXA-induced injury in parental tumors, and the knockdown of miR-31-5p expression enhanced sensitivity to OXA in resistant cells. 

Figure 5H determines the tumor cells metastasis to lung by H&E stain. OXA treatment reduced parental cells metastasis to lung but not in resistant cells. After the overexpression of miR-31-5p promoted tumor cells metastasis. Importantly, co-treatment with OXA and miR-31-5p mimic in LoVo cells OXA cannot inhibit tumor cells metastasis to lung. Moreover, lung metastasis was attenuated by injecting the miR-31-5p inhibitor in the OR-LoVo inhibitor and OR-LoVo inhibitor OXA groups. Accordingly, our in vitro and in vivo results indicate that OR-LoVo cells are resistant to OXA as a result of increasing the expression of miR-31-5p, which decreases the expression of LATS2 and its downstream proteins p21 and p27, induces cell proliferation, causing the tumor to become malignant, and leads to lung metastasis.

### 2.6. FOXC1 mRNA and Protein Expression in Colorectal Cancer

**Figure 6:**
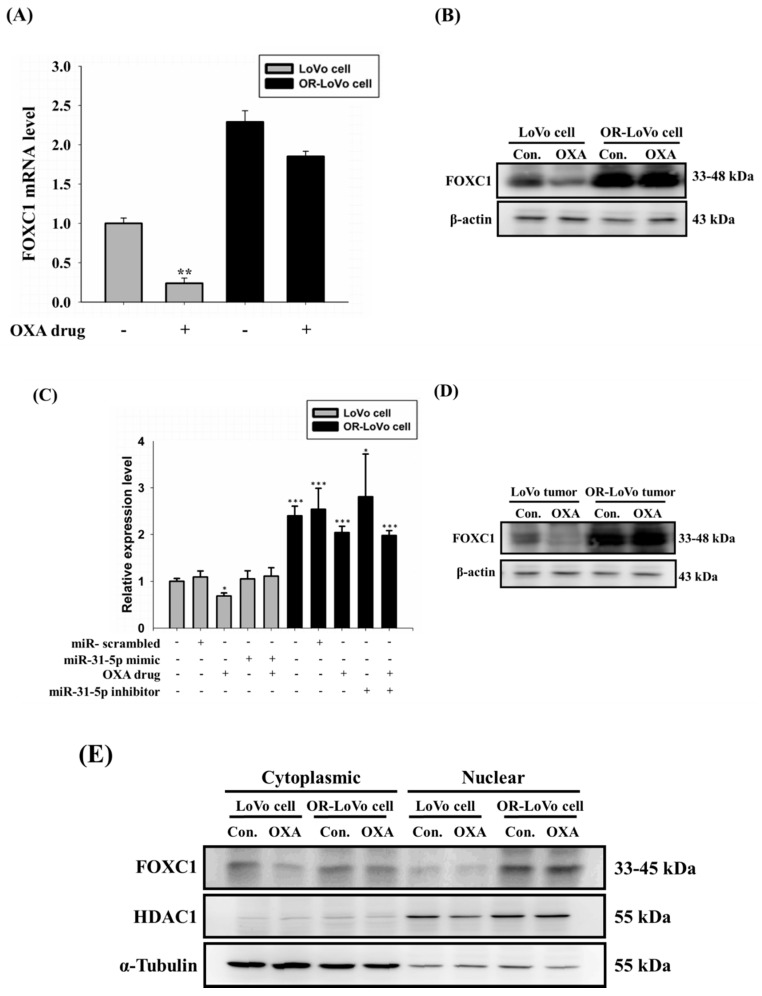
Experiments to identify the role of FOXC1 in colorectal cancer cells. (**A**) The FOXC1 mRNA level after treatment with or without OXA (45 μM) in LoVo and OR-LoVo cells in vitro. (**B**) Protein expression of FOXC1 following treatment with or without OXA (45 μM) in LoVo and OR-LoVo cells in vitro. (**C**) FOXC1 mRNA expression in the tumor formation assay in vivo. (**D**) Protein expression of FOXC1 following treatment with or without OXA in LoVo and OR-LoVo tumors in vivo. (**E**) FOXC1 nuclear translocation in LoVo cells and OR-LoVo cells in vitro. * *p* < 0.05 vs. LoVo cells control group; ** *p* < 0.01 vs. LoVo cells control group; *** *p* < 0.01 vs. LoVo cells control group.

Our previous results show that OR-LoVo cells acquire OXA resistance by upregulating miR-31-5p; thus, we aimed to identify the transcription factor that could induce miR-31-5p expression. We used the JASPAR database to predict transcription factors that bind to the miR-31 promoter. We found Forkhead box C1 (FOXC1), which is one of the transcription factors for miR-31-5 and also related to cancer cell proliferation [38]. Our results show that FOXC1 mRNA and its protein expression in OR-LoVo cells were both higher compared with those in LoVo cells. Treatment of LoVo cells with OXA decreased the FOXC1 mRNA and protein expression compared with non-treated cells (Figure 6A,B). Next, we examined FOXC1 expression in xenograft tumors. The FOXC1 mRNA level was increased in the OR-LoVo control group compared with that in the LoVo control group. The LoVo OXA group expressed a lower level of FOXC1 mRNA compared with that in the LoVo control group. The overexpression or knockdown of miR-31-5p had no significant effect on the FOXC1 level in vivo (Figure 6C). The protein expression of FOXC1 was evaluated following OXA in LoVo and OR-LoVo tumor tissue. The OR-LoVo tumor tissue expressed higher FOXC1 protein level compared with that in LoVo tumor tissue. The FOXC1 protein level in the LoVo OXA group was decreased compared with that in the LoVo control group. (Figure 6D). Then, we used cytoplasmic and nuclear protein isolation to evaluate the nuclear translocation of FOXC1 in LoVo and OR-LoVo cells. We found that FOXC1 could translocate from the cytoplasm to the nucleus in OR-LoVo cells but not in LoVo cells (Figure 6E). These data suggest that miR-31-5p is a downstream gene of FOXC1.

### 2.7. Binding of the FOXC1 Transcription Factor to the miR-31 Promoter Induces High miR-31-5p Expression

**Figure 7:**
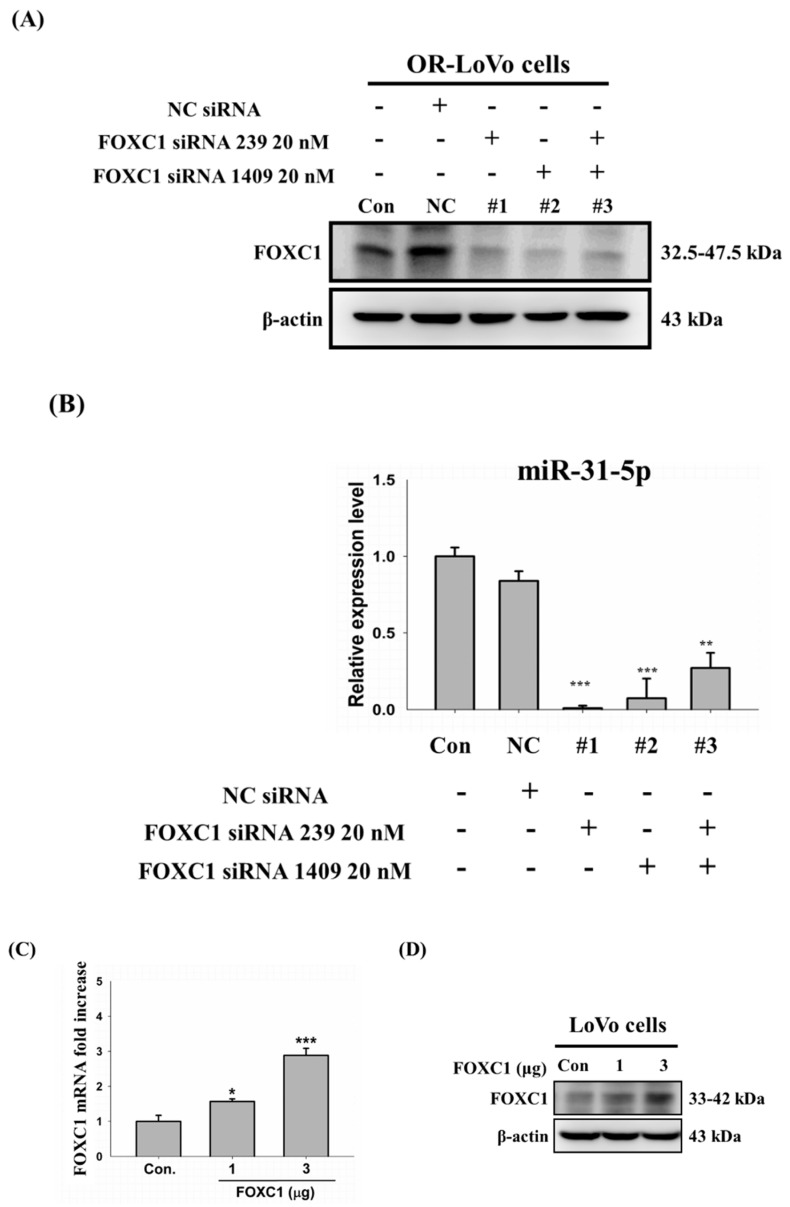

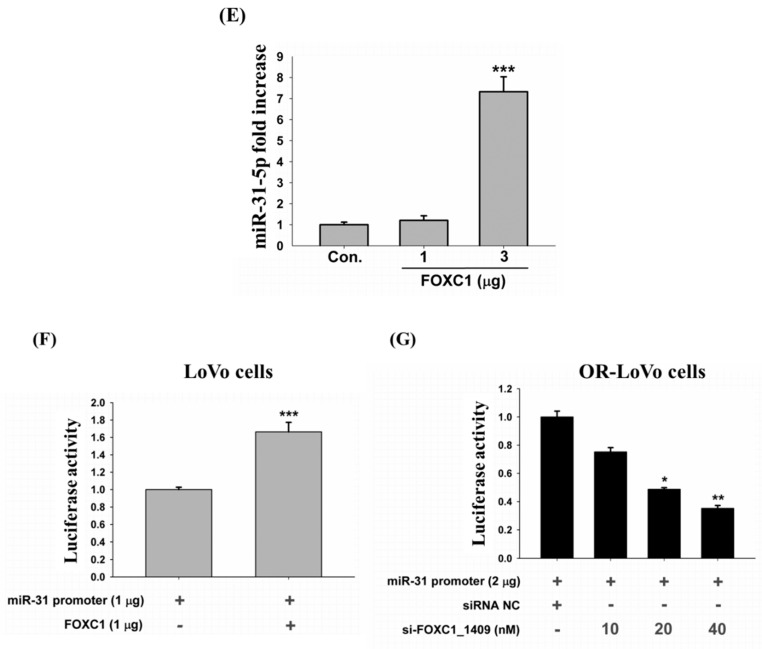
FOXC1 transcription factor binds to the miR-31 promoter and induces high miR-31-5p expression. (**A**) FOXC1 protein levels after the transfection of two different FOXC1 siRNAs in OR-LoVo cells. (**B**) MiR-31-5p levels in FOXC1-siRNA-transfected OR-LoVo cells. (**C**) FOXC1 mRNA expression following transfection of two doses of the FOXC1 overexpression construct (1 and 3 μg) in LoVo cells. The fold increase is calculated by the ratio FOXC1/GAPDH, being GAPDH snRNA constitutively expressed (**D**) FOXC1 protein levels resulting from two doses of the FOXC1 overexpression construct (1 and 3 μg) in LoVo cells. (**E**) MiRNA-31-5p expression resulting from two doses of the FOXC1 overexpression construct (1 and 3 μg) in LoVo cells. The fold increase is calculated by the ratio miR-31-5p/U6, being U6 snRNA constitutively expressed. (**F**) The luciferase reporter assay confirmed that LoVo cells were co-transfected with the miR-31 promoter (1 μg) and FOXC1 overexpression construct (1 μg). (**G**) The luciferase reporter assay confirmed that OR-LoVo cells were co-transfected with the miR-31 promoter (2 μg) and various concentrations of FOXC1-siRNA 1409 (0, 10, 20, and 40 nM). * *p* < 0.05 vs. control group; ** *p* < 0.01 vs. control group; *** *p* <0.001 vs. control group.

Accordingly, we aimed to confirm that FOXC1 is a direct transcription factor of miR-31-5p. FOXC1 expression in OR-LoVo cells could successfully be knocked down by transfection with FOXC1-siRNA (239, 1409, or both) (Figure 7A). The qRT-PCR data show that the expression of miR-31-5p decreased in OR-LoVo cells expressing FOXC1-siRNA compared with that in control cells (Figure 7B). On the other hand, we overexpressed FOXC1 by transfecting two different quantities of the FOXC1 construct (1 and 3 μg) in LoVo cells. FOXC1 mRNA expression increased in a dose-dependent manner in LoVo cells (Figure 7C). The same result was observed by Western blot (Figure 7D). The results of qRT-PCR detection indicate that the expression of miR-31-5p was significantly increased as a result of the overexpression of FOXC1 (3 μg) in LoVo cells compared with control cells (Figure 7E). Moreover, the luciferase activity of LoVo cells co-transfected with the miR-31 promoter (1 μg) and FOXC1 (1 μg) was significantly higher than that in LoVo cells transfected with only the miR-31 promoter (1 μg) (Figure 7F). In addition, the luciferase activity of OR-LoVo cells co-transfected with the miR-31 promoter (2 μg) and various concentrations of FOXC1-siRNA significantly decreased in a dose-dependent manner (Figure 7G).

## 3. Discussion

The role of miR-31 in cancers is still not clear. According to previous studies on different cancers, such as colorectal cancer (CRC) [3,24,39,40,41,42], lung cancer [22], and esophageal squamous cell carcinoma [23], miR-31 can act as an onco-microRNA. However, in breast cancer [43] and gastric cancer [44], miR-31 can act as a tumor suppressor gene. Importantly, we reveal a new function of miR-31 that is related to chemosensitivity, and it could thus be considered to be an onco-miRNA in CRC. In this study, we speculate that miR31 might have specific functions in each type of malignancy through several mechanisms, including cell proliferation, tumor growth, and metastasis, and it might have different roles in different tumor types. Additionally, clinical studies have indicated that high miR-31-5p expression is strongly related to a shorter progression-free survival (PFS) in all CRC patients with the wild-type gene who are treated with anti-epidermal growth factor receptor (EGFR) therapeutic agents [39]. In this study, we show that tumor size was significantly reduced after inhibiting miR-31-5p in a xenograft tumor model. This suggests that the function of miR-31-5p in human colorectal tumorigenesis may offer a new strategy for future colorectal cancer therapy. Moreover, our results indicate that miR-31-5p is not only related to tumors but also regulates colorectal cancer cell sensitivity to the chemo drug by targeting LATS2. Accordingly, we reveal that miR-31-5p plays an important role in the development of CRC and ultimately induces tumor growth and resistance in cancer cells by directly inhibiting LATS2 expression. These results clarify the mechanism by which miR-31-5p regulates chemoresistance in colorectal cancer.

LATS2 is a tumor suppressor that acts by phosphorylating downstream genes, such as YAP and TAZ. LATS2 may inhibit cell and tumor growth, and the overexpression of LATS2 was found to significantly attenuate the role of oncogenic miRNA [23,45] and vice versa. The expression of LATS2 enhances cancer cell growth, angiogenesis, metastasis, and malignant transformation of oncogenic miRNAs [23,32,46,47,48,49]. In this study, we discovered that LATS2 is a direct downstream target gene of miR-31-5p, and we demonstrate that miR-31-5p expression is significantly downregulated in parental tumors and cells compared with its expression in OR-LoVo tumor tissues and cell lines. Importantly, we clarify the mechanism by which miR-31-5p expression not only decreases LATS2 expression and increases tumor growth but also enhances drug sensitivity. Moreover, the overexpression of miR-31-5p even reversed the chemoresistance function in resistant cells and tumors. 

Additionally, we also highlight a novel miR-31 transcription factor, Forkhead box C1 (FOXC1). Previous reports have shown that FOXC1 regulates many pathways related to organ development and function, and many literature sources have shown that FOXC1 plays a key role in tumor progression and cancer cell proliferation. The results of our study confirm that FOXC1 also regulates microRNA expression. Clinical studies have also found that the increased expression of FOXC1 is closely related to a poor prognosis in many cancer operations [7,38,50,51,52,53,54,55]. In line with this, we reveal a mechanism by which FOXC1 is related to chemoresistance and promotes tumor growth via the miR-31-5p/LATS2 pathway.

In this study, we demonstrated a novel chemoresistance mechanism in colorectal cancer by performing in vitro and in vivo experiments that decreased the expression of miR-31-5p and FOXC1, which was found to be a transcription factor of miR-31-5p. Additionally, we confirmed the resistance mechanism since the deletion of miR-31-5p effectively inhibited tumor growth and chemoresistance, whereas the overexpression of miR-31-5p induced growth and chemoresistance in both cancer cells and tumors. These results suggest that miR-31-5p may be a novel biomarker for detecting the degree of colorectal cancer malignancy. Moreover, miR-31-5p and its transcription factor can be considered as new therapy targets in cancer patients who develop resistance to chemo drugs in clinical trials.

## 4. Materials and Methods

### 4.1. Cell Culture and OXA-Resistant Cell Establishment

LoVo cells or OR-LoVo cells were cultivated in RPMI 1640 medium (Gibco^TM^, Invitrogen Corporation, France) containing 10% FBS (characterized fetal bovine serum; HyClone, Logan, UT, USA), and 1% penicillin (Invitrogen Corp., Carlsbad, CA, USA) and were then incubated in humidified air with 5% CO_2_ at 37 °C.

Oxaliplatin-resistant LoVo cells (OR-LoVo cells) were established from LoVo cells following the protocol in our previous study [10]. In this study, the drug-resistant colorectal cancer cell line was established by exposing a LoVo cell line to doses of 0–25 μM oxaliplatin (OXA), which was changed from IV-injected liquid medicine (Sanofi, Paris, France) obtained from Dr. Ming-Cheng Chen [10] to the purified drug (Sigma, St. Louis, MO, USA), in a dose-dependent manner. The IC_50_ (50% cell death) of OXA in the LoVo cell line was found to be 15 μM, and this concentration was used to induce 90% cell death. The surviving cells were recovered to 80% in a culture plate and then passaged in the same OXA concentration to increase the OXA dose. In this study, we selected a cell population whose survival was under 3-fold the OXA IC_50_ concentration (45 μM) and identified this population as the OXA-resistant LoVo cell line.

### 4.2. MTT (Thiazolyl Blue Tetrazolium Bromide) Assay

Cell viability was measured using the MTT [3-(4,5-dimethylthiazol-2-yl)-2,5-diphenyltetrazolium-bromide] assay (Sigma) after treatment. Cells (1 × 10^4^/well) were seeded in 96-well plates in 100 μL and were treated with drugs or not treated, followed by incubation for 24 h. Thereafter, 100 µL of MTT (0.5 mg/mL) was added to the cells, followed by incubation for 3 h. The blue MTT formazan crystals were then dissolved in 150 μL of DMSO. The absorbance at 570 nm was measured using an ELISA reader. Cell viability was expressed as a percentage compared with the control using the following formula: (treated−blank)/(untreated control−blank).

### 4.3. Microarray Array Analysis

Total RNA was extracted from the LoVo and OR-LoVo cell lines and then analyzed by miRNA Profiling miRNA Microarray Services (Service Code: 2h213102401; Human miRNA OneArray^®^). The fold change was calculated by comparing the expression level of miRNAs in the OR-LoVo cells with that of the parental LoVo cells using a log2 format.

### 4.4. Prediction of mRNA Targets

Three established miRNA target prediction programs (miRTarBase, miRDB, and TargetScanHuman) were used to predict the putative targets of miR-31-5p.

### 4.5. Plasmid, miR-31-5p Overexpression (Mimic) or Knockdown (Inhibitor), Scrambled miRNA (Mimic NC or Inhibitor NC), and siRNA by Transfection

Plasmid encoding cofilin wild-type, LATS2 (LTS kinase 2 in pmirGLO Dual-Luciferase miRNA Target Expression Vector, GENEWIZ Clone ID # B44213-1/K264868), and hsa-miR-31 promoter in pGL4.10 [luc2] Vector (GENEWIZ Clone ID # BB5871-1/A386224) were purchased from GENEWIZ (www.genewiz.com). FOXC1 (Forkhead box C1, Human FOXC1 Gene ORF cDNA Clone expression plasmid) was purchased from Sino Biological (www.sinobiological.com). Human FOXC1 siRNA 239, Human FOXC1 siRNA 1409, and NC siRNA were purchased from Topgen (www.topgenbio.com.tw). 

The cells were 50%–70% confluent at the time of transfection. The cells were transfected with the plasmid, miR-31-5p overexpression (mimic), or knockdown (inhibitor), scrambled miRNA (mimic NC or inhibitor NC), and siRNA using jetPRIME® (Polyplus Transfection Inc, Illkirch, France). After a 24 h transfection, the cells were incubated in humidified air with 5% CO_2_ at 37 °C and then used in subsequent experiments.

### 4.6. Western Blot Analysis

The total protein content from the cells was extracted in cell RIPA buffer containing 50 mM Tris–HCl, pH 7.5, 150 mM NaCl, 1% NP-40, 0.1% SDS, and 0.5% sodium deoxycholate, supplemented with a mixture of complete protease inhibitors and phosphatase inhibitors. After centrifugation, the protein in the supernatant was collected and stored at −20 °C. The protein samples were determined using the Lowry protein assay. Briefly, equal amounts (20–30 μg) of protein were heated after adding the appropriate amount of 5× sodium dodecyl sulfate polyacrylamide gel electrophoresis (SDS-PAGE) sample loading buffer (40% glycerol, 10% SDS, 5% 2-mercaptoethanol, 0.02% bromophenol blue, 250 mM Tris–HCl, pH 8.8). The samples were separated on 6%–15% SDS-PAGE and subsequently transferred onto nitrocellulose filters using the Bio-Rad electrotransfer system (Bio-Rad Laboratories, Munich, Germany). The blots were then incubated with specific primary antibodies (1:1000) overnight at 4 °C, washed three times with TBST, and incubated with the appropriate secondary antibody for 1 h at room temperature. β-Actin, LATS2, Ki-67, α-SMA (Abcam, Cambridge, United Kingdom), ERK1 (BD Biosciences), p-Akt, p-ERK1/2, Akt, p21, p27, α-tubulin, HOAC1, and FOXC1 (Santa Cruz Biotechnology) primary antibodies were used. Finally, the blots were developed using a custom-made ECL detection system.

### 4.7. RNA Preparation and Quantitative RT-PCR Analysis

Total RNA from LoVo cells or OR-LoVo cells was extracted using the RNA Isolation System Quick-RNA™ MiniPrep kit (Zymo Research, Irvine, CA, USA) according to the manufacturer’s directions. Sample cDNA was prepared from LoVo cells or OR-LoVo cells using the iScript™ cDNA synthesis kit. cDNA was prepared from LoVo or OR-LoVo cancer tumor tissue using the Mir-X miRNA First-Strand Synthesis Kit. The cDNA samples were used as templates with appropriate primer sets to perform real-time PCR using IQ SYBR Green Supermix (Bio-Rad). Relative quantification was achieved by normalization to the amount of GAPDH mRNA or U6. The primers for LATS2 were F: 5′-AAGAGCTACTCGCCATACGCCTTT-3′, R: 5′-AGCTTTGGCCATTTCTTGCTCCAG-3′. The primers for GAPDH were F: 5′-GAAATCCCATCACCATCTTCCAGG-3′, R: 5′-GAGCCCCAGCCTTCTCCATG-3′. The primers for FOXC1 were F: 5′ -CGGGTTGGAAAGGGATATTTA-3′, R: 5′-CAAAATGTTCTGCTCCTCTCG-3′. The primers for miR-31-5p were AGGCAAGATGCTGGCATAGCT. mRQ 3′ Primer (10 μM), U6 Forward Primer (10 μM), and U6 Reverse Primer (10 μM) were from the Mir-X miRNA First-Strand Synthesis Kit and SYBR qRT-PCR User Manual. All reactions were performed in triplicate.

### 4.8. Luciferase Reporter Assay

Luciferase reporter assays were performed according to the manufacturer’s instructions (Dual-Luciferase® Reporter Assay System; Promega cat. no. E1910). 

The Dual-Luciferase miRNA target expression vector contained either the wild-type LATS2 3′UTR sequence or was left empty. Luciferase activity assays for miR-NC or miR-31 target validation were performed 24 h after transfection. The relative luciferase activities were normalized by Renilla luciferase activities. Each sample was measured in triplicate, and the experiment was repeated at least three times.

The miR-31 promoter was synthesized and inserted into the pGL4.10 [luc2] vector. Luciferase activity assays were conducted for FOXC1, siRNA NC, and si-FOXC1 24 h after transfection. The relative luciferase activities were normalized by Renilla luciferase activities. Each sample was measured in triplicate, and the experiment was repeated at least three times.

### 4.9. TUNEL Assay

Cells (5000 cells/0.5 mL/well) were plated onto Millicell® EZ slides in triplicate in 8-well plates and incubated for 24 h. Next, according to the different groups, the cells were transfected and underwent treatment for 24 h. We detected apoptosis in LoVo cells and OR-LoVo cells using the In Situ Cell Death Detection Kit and Fluorescein (Roche, Basel, Switzerland) according to the manufacturer’s instructions. This method can detect the fragmented DNA ends of apoptotic cells. For the quantification of apoptosis cells, five microscopic fields were randomly selected at 200× magnification, and the average counts of TUNEL-positive cells were calculated.

### 4.10. Subcutaneous Tumor Xenograft Model

Six-week-old nude mice were approved by the Institutional Animal Care and Use Committee of China Medical University, Taichung, Taiwan, ROC (ethic code: 2016-172-1). The mice were acquired from BioLASCO Taiwan Company. The number of cells in the two cancer cell lines (LoVo cells and OR-LoVo cells) used to subcutaneously inject into nude mice was 3 × 10^6^. After three weeks, when the tumors reached an average volume of 300–400 mm^3^, the mice were randomly divided into 10 groups with 4 mice in each group. According to the experimental design, intratumoral injection of miR-agomir NC, miR-31-5p agomir, miR-antagomir NC, miR-31-5p antagomir, or treatment with OXA was administered every 3 days. The tumor size was measured with a caliper every 3 days. The tumor volume was calculated using the formula volume = length × width2/2. The animals were housed in an environment maintained at a temperature of 25 ± 2 °C and a 12 h dark–light cycle with the lights on from 8 a.m. to 8 p.m. The normal animal diet (AIN-76) was purchased from Young Li Company (Taipei, Taiwan). Water was provided ad libitum throughout the experiment. The mice were sacrificed at day 15 after the tumors reached an average volume of 300–400 mm^3^, and the tumors were separated for further analysis. Ethics approval and consent to participate: Six-week-old nude mice were approved by the Institutional Animal Care and Use Committee of China Medical University, Taichung, Taiwan, ROC. The mice were acquired from BioLASCO Taiwan Company. The animals were housed in an environment maintained at a temperature of 25 ± 2 °C and a 12 h dark–light cycle with the lights on from 8 a.m. to 8 p.m. The normal animal diet (AIN-76) was purchased from Young Li Company (Taipei, Taiwan). Water was provided ad libitum throughout the experiment.

### 4.11. Immunohistochemistry (IHC) Staining

The organs and tumors were removed, fixed with formalin, embedded with paraffin, and sectioned. IHC of LATS2 and Ki-67 was performed on 10 μm sections using antibodies from Abcam (Cambridge, UK). The tumor tissue samples were then stained with R.T.U. VECTASTAIN^®^ Anti-Mouse IgG/Rabbit IgG/Goat IgG (Vector Laboratories, Cat. No. PK-7800) and ImmPACT™ DAB Peroxidase Substrate (Vector Laboratories, Cat. No. SK-4105) according to the manufacturer’s instructions.

### 4.12. Statistical Analysis

Each of our experiments was performed at least three times in an independent experiment. Data are expressed as the mean ± standard error. The relationship between two variables and the results obtained by real-time quantitative RT-PCR was analyzed using Student’s *t*-test. Multiple sets of comparisons were analyzed using one-way ANOVA. Statistically significant changes are indicated (* *p* < 0.05, ** *p* < 0.01, and *** *p* < 0.001).

## 5. Conclusions

**Figure 8:**
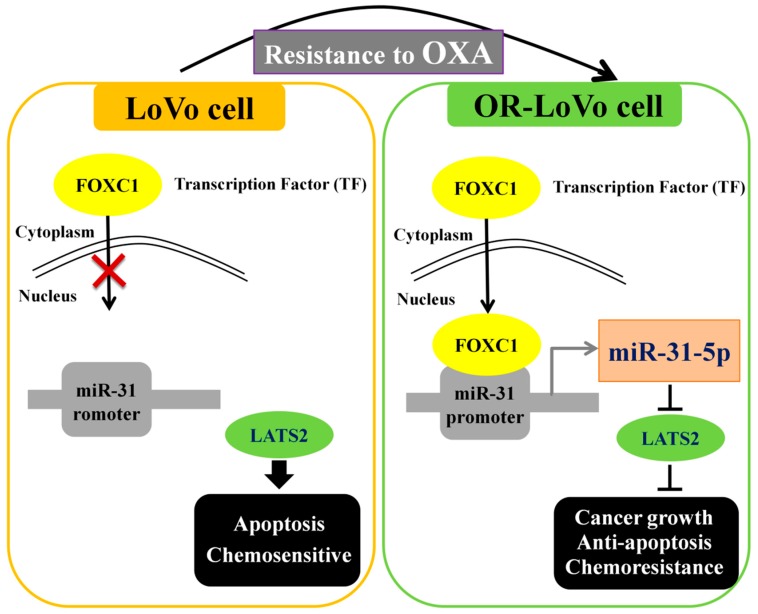
Schematic representation of the entire signaling pathway. The FOXC1 transcription factor regulates miR-31-5p expression in LoVo cells or OR-LoVo cells. The low nuclear localization of FOXC1 causes low miR-31-5p expression and high LATS2 expression, leading to apoptosis and increased OXA-based chemosensitivity in LoVo cells. By contrast, high miR-31-5p expression regulates the chemoresistance to OXA after FOXC1 binds to the miR-31 promoter of miR-31-5p, which targets LATS2, leading to cancer growth and suppression of apoptosis in OR-LoVo cells.

Our results show that the FOXC1 transcription factor regulates miR-31-5p expression and downstream genes in two cell lines. The low nuclear localization of FOXC1 causes low miR-31-5p expression and high LATS2 expression, leading to apoptosis and increased OXA-based chemosensitivity in LoVo cells. By contrast, high miR-31-5p expression regulates the chemoresistance of OXA after FOXC1 binds to the miR-31 promoter of miR-31-5p, which targets LATS2, leading to cancer growth and suppression of apoptosis in OR-LoVo cells (Figure 8). Importantly, our studies reveal a novel mechanism for LoVo cells’ resistance to OXA. In this mechanism, the upregulated expression of FOXC1 leads to expression of the miR-31-5p transcript, which induces cell proliferation by suppressing the expression of an anti-tumor gene, LATS2. These results suggest that miR-31-5p could be an essential target for treating drug resistance in CRC patients, and miR-31-5p could be used as a novel biomarker for the development of drug resistance in patients with CRC. Moreover, the FOXC1/miR31-5p/LATS2 drug-resistance mechanism may provide a new therapeutic strategy for CRC in clinical trials.

## Supplementary Materials

The following are available online at https://www.mdpi.com/2072-6694/11/10/1576/s1, Figure S1: Nude mice weight changed after tumor cells injection.
