# Supplementary material for: FOXC1 Regulation of miR-31-5p Confers Oxaliplatin Resistance by Targeting LATS2 in Colorectal Cancer
Source: Cancers (Basel). 2019 Oct 16;11(10):1576. doi: 10.3390/cancers11101576 (PMC6827018; doi:10.3390/cancers11101576)

Supplementary Materials: FOXC1 Regulation of miR-31-5p Confers Oxaliplatin Resistance by Targeting LATS2 in Colorectal Cancer

Hsi-Hsien Hsu, Wei-Wen Kuo,Hui-Nung Shih, Sue-Fei Cheng, Ching-Kuo Yang, Ming-Cheng Chen, Chuan-Chou Tu, Vijaya Padma Viswanadha, Po-Hsiang Liao and Chih-Yang Huang

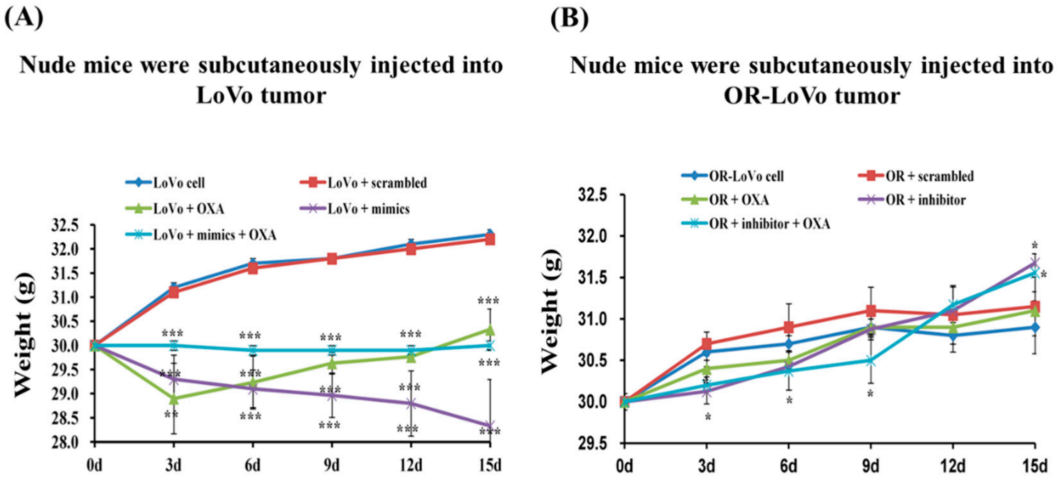

Figure S1. Nude mice weight changed after tumor cells injection. (**A**) The nude mouse weight was measured in five different groups: the LoVo cell group as the LoVo control group, the LoVo scrambled group treated with miR-agomir NC, the LoVo OXA group, the LoVo mimic group, and the LoVo mimic OXA group treated with or without OXA, miR-31-5p agomir, or both, administered every 3 days from day 0 to 15. (**B**) The nude mouse weight was measured in five different groups: the OR-LoVo cell group as the OR-LoVo control group, the OR-LoVo scrambled group treated with miR-antagomir NC, the OR-LoVo OXA group, the OR-LoVo inhibitor group, and the OR-LoVo inhibitor OXA group treated with or without OXA, miR-31-5p antagomir, or both, administered every 3 days from day 0 to 15.

For the western blotting data we used ImageQuant LAS 4000 mini (GE Healthcare Life Sciences), the readings/intensity ratio of each band we used standard/ exporsured 20 second.

KI-67

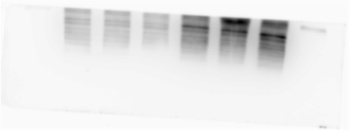

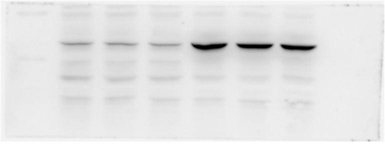

p-AKT

ERK1

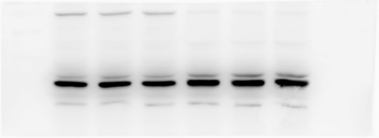

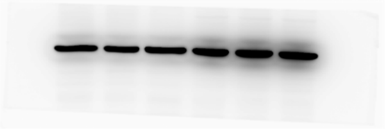

a-SMA

p-ERK

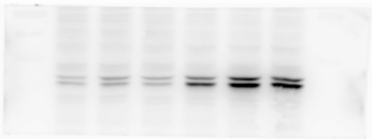

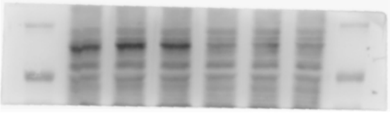

p21

AKT

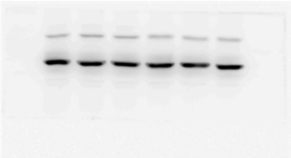

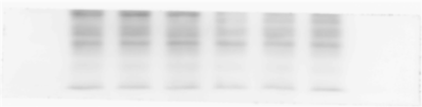

p27

LATS2

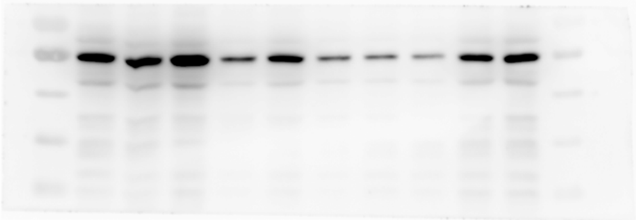

p21

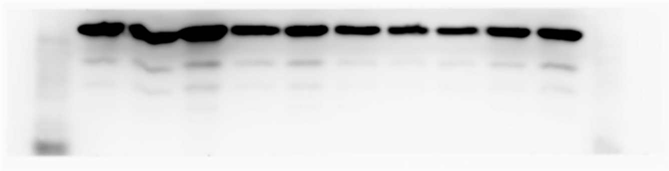

p27

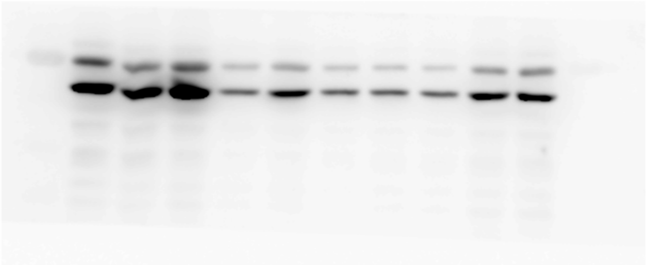

LATS2

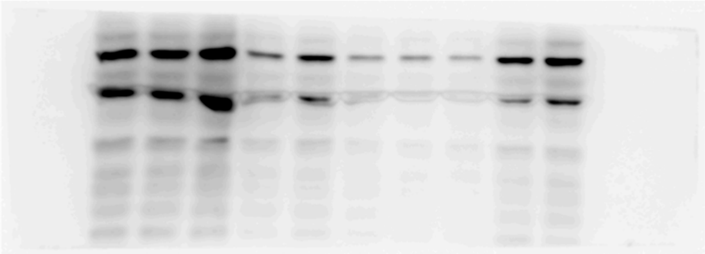

FOXC1

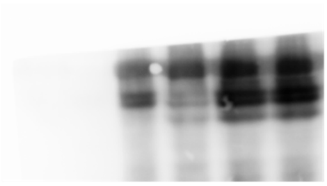

FOXC1

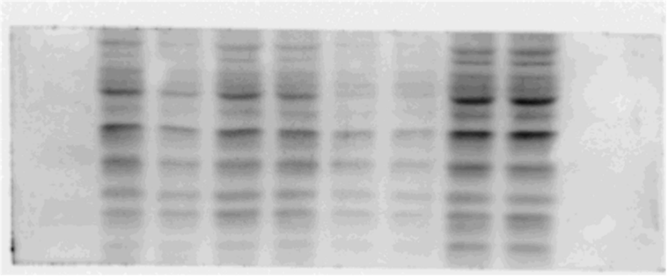

HDAC1

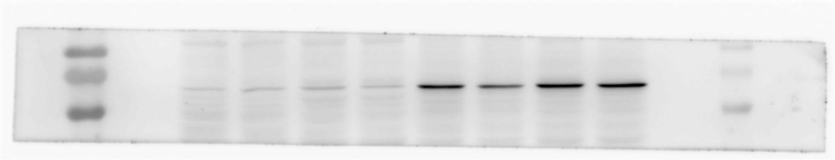

FOXC1

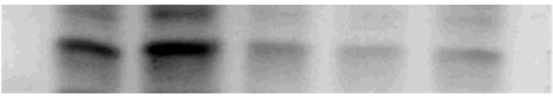

FOXC1

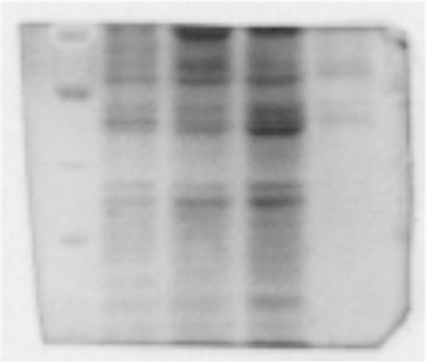

Supplement: Supplementary file 1 [file cancers-11-01576-s001.pdf]
